# Тестостерон и болезнь Альцгеймера

**DOI:** 10.14341/probl13136

**Published:** 2022-06-24

**Authors:** К. О. Кузнецов, Р. Р. Хайдарова, Р. Х. Хабибуллина, Е. С. Стыценко, В. И. Философова, И. Р. Нуриахметова, Э. М. Хисамеева, Г. С. Важоров, Ф. Р. Хайбуллин, Е. А. Иванова, К. В. Горбатова

**Affiliations:** Российский национальный исследовательский медицинский университет им. Н.И. Пирогова; Башкирский государственный медицинский университет; Первый Санкт-Петербургский государственный медицинский университет им. акад. И.П. Павлова; Санкт-Петербургский государственный педиатрический медицинский университет; Первый Санкт-Петербургский государственный медицинский университет им. акад. И.П. Павлова; Башкирский государственный медицинский университет; Башкирский государственный медицинский университет; Чувашский государственный университет им. И.Н. Ульянова; Башкирский государственный медицинский университет; Курский Государственный Медицинский Университет; Курский Государственный Медицинский Университет

**Keywords:** болезнь Альцгеймера, тестостерон, андрогены, андроген-депривационная терапия, когнитивные функции

## Abstract

Болезнь Альцгеймера (БА) является нейродегенеративным заболеванием, которое становится причиной деменции в половине случаев ее возникновения. БА, как правило, обнаруживается у людей старше 65 лет. Этиопатогенез заболевания является многофакторным и включает генетические факторы, нарушения питания, митохондриальную дисфункцию, окислительный стресс и старение. Половые гормоны оказывают важное влияние на развитие БА, о чем свидетельствует большая заболеваемость у женщин, чем у мужчин. Учитывая значительное влияние тестостерона (Т) на поддержание нормального функционирования головного мозга, настоящее исследование направлено на оценку влияния андроген-депривационной терапии, а также терапии тестостероном на риск развития и прогрессирования БА. Хотя между исследованиями существует некоторое клиническое несоответствие, андрогены оказывают значительное влияние на функцию головного мозга и полезны для пациентов с БА. Низкие уровни циркулирующих андрогенов следует рассматривать как существенный фактор риска развития БА и потери памяти. При сниженном уровне Т в плазме мужчин его введение способствует повышению когнитивной работоспособности и памяти, лечение следует начинать на ранней стадии заболевания. У мужчин и женщин с БА андрогены улучшают психическое состояние и замедляют прогрессирование заболевания, оказывая протективное действие. В будущем необходимо проведение исследований на большой популяции с учетом факторов индивидуальности и более конкретным подходом к оценке когнитивных функций и причинной-следственной связи введения Т при БА.

## Abstract (en)

Alzheimer’s disease (AD) is a neurodegenerative disease that causes dementia in half of the cases. Asthma is usually found in people over 65 years of age. The etiopathogenesis of the disease is multifactorial and includes genetic factors, nutritional disorders, mitochondrial dysfunction, oxidative stress, and aging. Sex hormones have an important influence on the development of AD, as evidenced by a higher incidence in women than in men. Considering the significant influence of T on the maintenance of normal brain function, the present study is aimed at evaluating the impact of androgen deprivation therapy (ADT), as well as testosterone therapy, on the risk of AD development and progression. Although there is some clinical inconsistency between studies, androgens have a significant effect on brain function and are beneficial for AD patients. Low levels of circulating androgens should be considered as a significant risk factor for the development of AD and memory loss. With a reduced level of T in the plasma of men, its administration improves cognitive performance and memory, treatment should be started at an early stage of the disease. In men and women with AD, androgens improve mental state and slow the progression of the disease, providing a protective effect. In the future, it is necessary to conduct studies on a large population, taking into account personality factors and a more specific approach to assessing cognitive functions and the causal relationship of T administration in AD.

## ВВЕДЕНИЕ

Болезнь Альцгеймера (БА) является нейродегенеративным заболеванием, которое становится причиной деменции в половине случаев ее возникновения [1]. БА, как правило, обнаруживается у людей старше 65 лет. Этиопатогенез заболевания является многофакторным и включает генетические факторы, нарушения питания, митохондриальную дисфункцию, окислительный стресс и старение [2]. БА характеризуется аномальным отложением бета-амилоида в нейронах с образованием внеклеточных бляшек, ответственных за дегенерацию нейронов [3], а также за дисфункцию синапсов [4].

Половые гормоны оказывают важное влияние на развитие БА, о чем свидетельствует большая заболеваемость у женщин, чем у мужчин [5]. В исследованиях, проведенных на клеточных культурах [6] и на животных моделях БА [7][8], было продемонстрировано, что уровень тестостерона (Т) тесно связан с эффективностью функционирования нейронов, а также способен снижать отложение бета-амилоида в головном мозге. Т стимулирует фагоцитоз микроглии, удаляя отложения бета-амилоида и ингибируя воспалительную реакцию [9]. На крысиной модели БА было показано, что Т предотвращает снижение когнитивных функций путем поглощения свободных радикалов, тем самым усиливая синаптическую пластичность [8][10], также Т регулирует биоэнергетику нейронов, повышая митохондриальную функцию [11], повышает антиоксидантную активность и предотвращает нейродегенеративные расстройства. Кроме того, Т снижает инсулинорезистентность [12], а также предотвращает процессы старения сосудов и нейронов, увеличивая активность eNOS и стимулируя экспрессию SIRT1 [13]. Т-опосредованное повышение экспрессии белка SYN приводило к улучшению поведенческих показателей и обучаемости в модели ускоренного старения мышей [14].

У мужчин низкие уровни Т в сыворотке крови коррелируют с риском развития БА [15]. Напротив, высокие уровни свободного Т в сыворотке крови обоих полов, по-видимому, оказывают протекторное действие в отношении развития БА [16]. J. Lee и соавт. обнаружили, что высокий уровень свободного Т у пожилых пациентов, которые были обследованы при помощи магнитно-резонансной томографии, коррелировал с более низким отложением бета-амилоида, а также с меньшей выраженностью когнитивных нарушений, в то время как свободный эстрадиол не оказывал значимого влияния на вышеуказанные параметры у обоих полов [16]. Это исследование показало, что Т оказывает наибольшую активность на ранней стадии патологического накопления бета-амилоида. Другие исследования показали, что низкий уровень Т в сыворотке крови у мужчин был связан с повышенным отложением бета-амилоида, что приводило к развитию БА [16][17] и синаптической дисфункции с последующим снижением когнитивных функций [4].

Цель исследования — учитывая значительное влияние Т на поддержание нормального функционирования головного мозга, настоящее исследование направлено на оценку влияния андроген-депривационной терапии (АДТ), а также терапии Т на риск развития и прогрессирования БА.

## ВЛИЯНИЕ ТЕСТОСТЕРОНА НА БОЛЕЗНЬ АЛЬЦГЕЙМЕРА

Наличие большого количества андрогенных рецепторов (АР) в головном мозге предполагает, что андрогены играют соответствующую физиологическую роль в функционировании нейронов. АР в основном локализуются в гипоталамусе и миндалевидном теле, которые играют важную роль в процессе обучения и запоминания, а также в конечном и спинном мозге [18]. Нейротрофический эффект Т заключается в активации АР и предотвращении отложения бета-амилоида на нейронах какза счет прямого действия, так и опосредованно, через действие метаболита 17β-эстрадиола [19]. Т улучшает энергетический обмен и снижает окислительный стресс в нейронах [20], а также понижает активность бета-секретазы-1 (BACE-1), которая участвует в образовании бета-амилоида [21]. Влияние Т на клеточную биоэнергетику является более выраженным, чем у других половых гормонов, включая прогестерон и эстрогены [22]. Влияние Т на нейроны является сложным и обуславливается его прямым действием в сочетании с действием его метаболитов.

Т может быть подвержен процессу ароматизации и преобразоваться в 17β-эстрадиол, превратиться в дигидротестостерон (ДГТ) под действием 5α-редуктазы, а андростендион под действием 3α-гидроксистероиддегидрогеназы (3α-ГСД) может быть преобразован в 3α-андростандиол (3α-диол), который обладает эстрогенным эффектом и активирует рецепторы гамма-аминомасляной кислоты (ГАМК). 17β-эстрадиол активирует рецепторы эстрогена (ЭР), тем самым потенцируя некоторые эффекты Т. Все вышеуказанные нейростероиды участвуют в регуляции активности нейронов [23].

Однако Т оказывает нейропротекторное действие независимо от его биотрансформации в эстрадиол [21], потенцируя анти-бета-амилоидный эффект и снижая гибель нейронов [24]. Метаболит 3α-диол представляет соответствующий интерес, поскольку является мощным нейростероидом, модулирующим рецептор ГАМК, оказывая противосудорожное действие и восстанавливая когнитивные функции [25]. Андростендиол активен в отношении рецепторов ГАМК и N-метил-d-аспартата (NMDA), которые ответственны за память, обучаемость и психоз. Примечательно, что 5α-андростан,3β,17β-диол (3β-диол) активирует ЭР, а не АР. На РНК NMDA-рецепторов также влияют уровни соматотропного гормона и инсулиноподобного фактора роста-1 (ИФР-1), которые увеличивают их экспрессию (рис. 1) [26].

**Figure:**
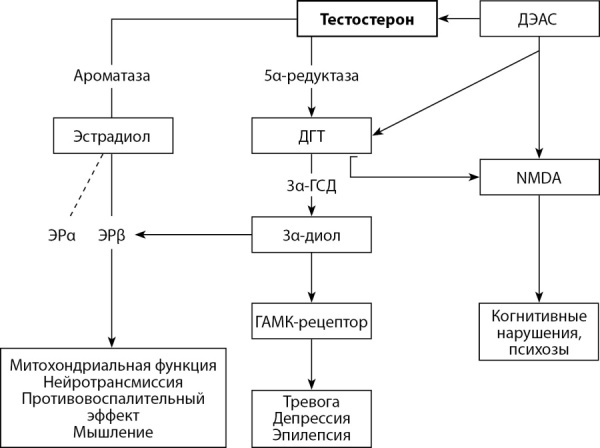
Рисунок 1. Воздействие тестостерона и его метаболитов на головной мозг.Примечание: ДЭАС — дегидроэпиандростерон-сульфат; ДГТ — дигидротестостерон; 3α-ГСД — 3α-гидроксистероиддегидрогеназа; 3α-диол — 3α-андростандиол; NMDA — N-метил-d-аспартат; ГАМК — гамма-аминомасляная кислота; ЭР — рецептор эстрогена.Figure 1. Effects of testosterone and its metabolites on the brain.

Т влияет на процесс познания, улучшая синаптическую пластичность [10], а также увеличивая количество интактных клеток и плотность дендритных шипиков в области гиппокампа [7]. В случае низкого уровня Т в сыворотке крови многие биохимические и метаболические процессы в головном мозге нарушаются (рис. 1).

По результатам метаанализа было установлено, что низкий уровень Т в плазме был достоверно связан с повышенным риском развития БА, и его следует рассматривать как фактор риска ухудшения когнитивных функций у пожилых мужчин [15].

## РОЛЬ ЭСТРАДИОЛА

В эксперименте на мышах было продемонстрировано, что эстрадиол играет важную роль в регуляции эндогенного нейрогенеза, синаптической пластичности и когнитивных функций на ранней стадии БА [27], а также замедляет снижение когнитивных функций у молодых мышей [28]. У женщин эстрогены выполняют защитную функцию, направленную на замедление нейродегенерации, а с наступлением менопаузы падение их уровня в плазме крови является определяющим фактором развития БА. У женщин в менопаузе андрогены и глобулин, связывающий половые гормоны (ГСПГ), постепенно снижаются [29][30]. Эстрадиол является продуктом ароматизации Т во внегонадной ткани и регулируется экспрессией ароматазы [31]. Средний уровень андрогенов в плазме крови значительно снижается в процессе старения. Плазменный уровень общего и свободного Т в диапазоне от 65 до 74 лет по сравнению с диапазоном от 18 до 24 лет колеблется от 1,8 до 0,66 нмоль/л и от23,61 до 10,81 пмол/л соответственно. Дегидроэпиандростерон-сульфат (ДЭАС) и андростендион также снижаются на одну треть [29].

Тем не менее открытым остается вопрос об эффективности введения эстрогенов женщинам в менопаузе для предотвращения развития БА. Хотя некоторые исследования установили снижение заболеваемости БА и деменцией у женщин, принимающих терапию эстрогенами [32–34], ряд других исследований не выявил положительного эффекта [35][36]. Недавнее исследование, проведенное в большой популяции, состоящей из 84 739 женщин в постменопаузе, показало, что систематическое введение эстрогенов имело корреляцию с увеличением заболеваемости БА [37]. Плацебо-контролируемые исследования показали повышенный риск заболеваемости деменцией у женщин, которые получали конъюгированный лошадиный эстроген, в то время как прогестерон не оказывал никакого влияния [38]. A.M. Tolppanen и соавт. оценивали распространенность использования системных эстрогенов у женщин с БА и без БА. По результатам исследования авторы невыявили достоверных различий между сравниваемыми группами [39].

Заболеваемость БА связана не только с активностью эстрогена, но и с уровнем андрогенов в плазме крови, что объясняет более высокий риск развития БА у женщин по сравнению с мужчинами.

Эстрогены оказывают не противоположное, но различное влияние на мозг мужчин и женщин [40]. Они синтезируются не только в репродуктивных тканях, но и в головном мозге с последующим воздействием на ЭР [41]. Тиболон является синтетическим гормональным средством, которое обладает эстрогенной, андрогенной и прогестагенной активностью и имеет нейропротекторный эффект [42]. На сегодняшний день имеется не много исследований, посвященных оценке влияния тиболона на центральную нервную систему, однако все они подтвердили его положительное действие на процессы обучения и запоминания [43–45]. Эти исследования показывают, что комбинация Т с эстрогеном, вероятно, может иметь значение при терапии БА. Кроме того, нейропротекторное действие эстрогенов может быть связано с влиянием на сигнальную систему ИФР-1 [46], которая также играет немаловажную роль в патогенезе БА.

## МАТЕРИАЛЫ И МЕТОДЫ

Нами был проведен электронный поиск публикаций в базах данных PubMed, Scopus, MEDLINE и Google Scholar. В исследование включались статьи, опубликованные с 2000 г. по настоящее время. Критериями поиска было наличие слов «androgen deprivation therapy» AND «Alzheimer’s disease»; «dementia»; «Alzheimer’s disease»; «testosterone therapy» AND «Alzheimer’s disease» в ключевых словах, аннотациях и названиях статей. Разногласия между авторами разрешали путем консенсуса.

## РЕЗУЛЬТАТЫ

Нами было отобрано 20 статей по влиянию АДТ на когнитивные нарушения и развитие БА (табл. 1) и 17 статей, посвященных влиянию терапии Т на БА и когнитивные функции (табл. 2). Критерием включения было отсутствие онкологического анамнеза до постановки диагноза рака предстательной железы (РПЖ).

**Table:** Таблица 1. Влияние АДТ на когнитивные нарушения и развитие БАTable 1. The effect of androgen deprivation therapy on cognitive impairment and the development of Alzheimer's disease Примечание: АДТ — андрогенная депривационная терапия; БА — болезнь Альцгеймера; РПЖ — рак предстательной железы; ИБС — ишемическая болезнь сердца.

Исследование	Характеристика выборки	Средний возраст	Метод исследования	Время наблюдения	Результаты
J.H. Hong и соавт., 2020 [47]	24 464 мужчины с РПЖ	АДТ — 74,1Без АДТ — 71,0	Когортное исследование	4,98 года	АДТ имела значимую корреляцию с риском снижения когнитивных функций
W.K. Huang и соавт., 2020 [48]	23 651 мужчина с РПЖ	73	Когортное исследование	-	АДТ имела значительную корреляцию с повышенным риском развития деменции или БА
R. Jayadevappa и соавт., 2019 [49]	154 089 мужчин с РПЖ	76	Ретроспективное исследование	8,3 года	Проведение АДТ было связано с последующей диагностикой БА или деменции
A. Krasnova и соавт., 2020 [50]	100 414 мужчин с РПЖ	73	Обсервационное исследование	6 мес	АДТ коррелировала с более высоким риском развития деменции и БА
P. Jarzemski и соавт., 2019 [51]	100 пациентов, перенесших простатэктомию	50–77	Обсервационное исследование	-	Комплексная терапия РПЖ приводила к когнитивным расстройствам
D. Robinson и соавт., 2019 [52]	25 967 мужчин с РПЖ;121 018 — контроль;	76,5	Популяционное когортное исследование	4 года	Исследование не выявило повышенного риска развития БА у мужчин, получающих АДТ
B.S. Tae и соавт., 2019 [53]	35 401 пациент с РПЖ из базы Службы национального страхования Южной Кореи	70	Проспективное исследование	7 лет	АДТ имеет корреляцию с повышенным риском развития когнитивной дисфункции
C. Nguyen и соавт., 2018 [54]	201 797 мужчин с РПЖ (94 528 пациентов получали АДТ)	66	Проспективное исследование	19 лет	АДТ имела корреляцию с более высоким риском переломов костей, сахарного диабета, деменции и ИБС
S. Marzouk и соавт., 2018 [55]	81 мужчина с РПЖ	69	Когортное исследование	1 год	АДТ не имела корреляции со снижением когнитивной функции при неметастатическом РПЖ
R. Deka и соавт., 2017 [56]	45 218 мужчин с РПЖ	Не сообщается	Обсервационное исследование	6,8 года	Статистически значимого увеличения риска развития деменции или БА на фоне АДТ не выявлено
S.H. Baik и соавт., 2017 [57]	109 815 мужчин с РПЖ	67	Анализ выживаемости	-	Риск развития БА и деменции не был связан с продолжительностью АДТ
S.M. Alibhai и соавт., 2017 [58]	77 пациентов с РПЖ + проведение АДТ;82 пациента с РПЖ без АДТ;82 пациента — контроль	68,9	Исследование «случай-контроль»	3 года	Проведение АДТ не имело корреляции со снижением когнитивных функций
L.T. Kao и соавт., 2017 [59]	755 мужчин с РПЖ	74,2	Проспективное исследование	5 лет	Между развитием БА и АДТ корреляции не выявлено
B. Gunlusoy и соавт., 2017 [60]	78 мужчин с метастатическим РПЖ;	67,1;	Проспективное исследование	1 год	АДТ не оказывала влияния на когнитивные функции (речь, память, умственная пластичность)
78 пациентов — контроль	68,6
K.T. Nead и соавт., 2017 [61]	9 455 мужчин с РПЖ	69,9	Обсервационное исследование	3,4 года	АДТ имела корреляцию с повышенным риском деменции
F. Khosrow-Khavar и соавт., 2017 [62]	30 903 мужчины с РПЖ	70,7	Проспективное исследование	4,3 года	АДТ не имела корреляции с повышенным риском развития деменции
L.M. Wu и соавт., 2016 [63]	19 пациентов с РПЖ, получающих АДТ;	67,5;	Ретроспективное исследование	-	Пациенты, получающие АДТ, более склонны к развитию специфических когнитивных и нейроповеденческих расстройств
20 — контроль	70,0
S.D. Chung и соавт., 2016 [64]	1335 пациентов с РПЖ;4005 — контроль	72,2	Ретроспективное исследование	5 лет	Применение АДТ при РПЖ не имело корреляции с повышенным риском развития БА или болезни Паркинсона
K.T. Nead и соавт., 2016 [65]	16 888 мужчин с РПЖ	70,0	Ретроспективное исследование	2,7 года	АДТ увеличивала риск развития БА в общей популяции
B.D. Gonzalez и соавт., 2015 [66]	58 пациентов с РПЖ + проведение АДТ;	67,3	Сравнительное исследование	5 лет	Проведение АДТ демонстрирует нарушение когнитивных функций через 6 и 12 мес
84 пациента с РПЖ без АДТ;	67,7
88 пациентов — контроль	69,1

**Table:** Таблица 2. Влияние терапии тестостероном на БА и когнитивные нарушенияTable 2. Effects of Testosterone Therapy on Alzheimer's Disease and Cognitive Impairment Примечание: БА — болезнь Альцгеймера; РКИ — рандомизированное контролируемое исследование; Т — тестостерон; ЛКР — легкие когнитивные расстройства; ГнРГ — гонадотропин-рилизинг-гормон; ADAS-cog (Alzheimer disease assessment scale-cognitive) — когнитивная шкала оценки болезни Альцгеймера; MMSE (Mini-Mental State Examination) — краткая шкала оценки психического статуса; CDT (Clock Drawing Test) — тест «Рисование часов».

Исследование	Характеристика выборки	Средний возраст	Метод исследования	Проводимая терапия	Длительность терапии	Результат
S.M. Resnik и соавт., 2017 [74]	788 мужчин с нарушением половой функции	65	РКИ	Т-гель с дозой для поддержания физиологического уровня в плазме	4 года	Отсутствует корреляция с улучшением памяти и других когнитивных функций
E.J. Wahjoepramono и соавт., 2016 [75]	44 мужчины	≥50	РКИ	Т-гель 50 мг	24 нед	Значительное улучшение когнитивных функций
G. Huang и соавт., 2016 [76]	308 мужчин с низким Т	60	РКИ	Т-гель 7,5 г 1%	36 мес	Введение Т не улучшало когнитивные функции
P.R. Asih и соавт., 2015 [77]	44 пожилых мужчины	61±7,7	РКИ	Трансдермальный Т (50 мг/сут)	24 нед	Значительное повышение андрогенов в плазме. Отсутствует изменение уровня бета-амилоида в плазме
M.M. Cherrier и соавт., 2015 [78]	351 мужчина.37 с ЛКР и низким Т	70,5±8,2	РКИ	Т-гель (от 50 до 100 мг/сут)	3 мес	Умеренное улучшение вербальной памяти и уменьшение выраженности симптомов депрессии
S.E. Borst и соавт., 2014 [79]	60 мужчин с гипогонадизмом	70,8	РКИ	Т-энантат (125 мг/нед)	12 мес	Небольшое снижение симптомов депрессии и улучшение зрительно-пространственного познания
L.A. Young и соавт., 2010 [80]	26 молодых мужчин;	25–35	РКИ	Агонист ГнРг, Т-гель 75 и 100 мг	6 нед	Т улучшал пространственное познание, в то время как эстрадиол коррелировал со снижением памяти
62 пожилых мужчины	60–80
M.H. Emmelot-Vonk и соавт., 2008 [81]	237 здоровых мужчин с низким уровнем Т	60–80	РКИ	Т-ундеканоат 80 мг	6 мес	Когнитивные функции и минеральная плотность костной ткани не изменились
C. Vaughan и соавт., 2007 [82]	65 здоровых мужчин	-	РКИ	200 мг Т каждые 2 недели + 5 мг финастерида в день (T + F) или плацебо	36 мес	Клинически значимого эффекта на когнитивные функции не выявлено
P.M. Maki и соавт., 2007 [83]	15 здоровых мужчин	66–87	РКИ	Т-энантат (200 мг каждые 2 недели)	3 мес	Снижение вербальной памяти
M.M. Cherrier и соавт., 2007 [84]	57 здоровых мужчин	67±11	РКИ	Т-энантат 50, 100 или 300 мг/нед	6 нед	Существенных изменений памяти не выявлено
P.H. Lu и соавт., 2006 [85]	16 мужчин с БА легкой степени	-	РКИ	Т-гель (75 мг)	24 нед	Улучшение качества жизни у пациентов с БА. Т оказывал минимальное влияние на познание
M.T. Haren и соавт., 2005 [86]	76 здоровых мужчин	60	РКИ	Т-ундеканоат 80 мг два раза в день	12 мес	Не выявлено влияния на визуально-пространственные тесты, шкалы настроения и качество жизни
A.M. Kenny и соавт., 2004 [87]	11 мужчин со снижением когнитивных функций	80±5	РКИ	200 мг Т каждые 3 недели	12 нед	Нет существенных изменений в поведении и когнитивных функций
R.S. Tan и соавт., 2003 [88]	36 мужчин с БА;10 гипогонадных мужчин	-	РКИ	Внутримышечный Т 200 мг каждые 2 недели	12 мес	Отмечено значительное улучшение показателей ADAS-cog, CDT и MMSE у пациентов, получавших лечение
D.B. O’Connor и соавт., 2001 [89]	30 здоровых мужчин и 7 гипогонадных	-	РКИ	200 мг Т-энантата еженедельно	8 недель	Повышенный уровень Т оказывает дифференциальное влияние на когнитивные функции, подавляя пространственное мышление при одновременном улучшении речи
M.M. Cherrier и соавт., 2001 [90]	25 здоровых мужчин	-	РКИ	Т-энантат 100 мг в неделю	6 нед	Кратковременное введение Т улучшает когнитивные функции

## АНДРОГЕННАЯ ДЕПРИВАЦИОННАЯ ТЕРАПИЯ И РИСК РАЗВИТИЯ БА

Двадцать исследований, проведенных на больших когортах пациентов, изучали влияние АДТ на риск развития БА или деменции [47–66]. Исследования обобщены в таблице 1. Большинство (13 исследований) установили значительную корреляцию между АДТ и риском развития БА и других когнитивных нарушений [47–51][53][54][58][60][61][63][65][66], в то время как другие не выявили никакой взаимосвязи [52][55–59][62][64]. Продолжительные исследования показали, что у мужчин с РПЖ, получавших АДТ, уровень Т в плазме снижался, в то время как уровень бета-амилоида увеличивался [17][67]. Результаты систематических обзоров показывают, что мужчины с РПЖ, получающие АДТ, имеют более высокий риск развития когнитивных нарушений и деменции [68], а также симптомов депрессии [69][70].

Противоречивые результаты, полученные в результате исследований, отражают сложность оценки влияния АДТ на функцию головного мозга и риск развития БА. S.H. Baik и соавт. на протяжении 5,5 года исследовали популяцию, состоящую из1 238 879 пациентов, из которых 35% подвергались хирургической или химической АДТ, и они не обнаружили корреляции между АДТ и БА [57]. Однако в исследовании не учитывались: использование антиандрогенов, семейный анамнез по БА, вредные привычки, а также информация о развитии РПЖ и уровни биомаркеров. Также не принималась во внимание рутинная терапия РПЖ. Кроме того, не проводились тесты для оценки когнитивных функций. S.D. Chung и соавт. исследовали большую популяцию и продемонстрировали, что АДТ не коррелирует с риском развития БА и болезни Паркинсона [64]. Тем не менее никакой конкретной информации о пациентах в исследовании не приводится, отсутствует учет факторов индивидуальности.

Исследование K.T. Nead и соавт. [70], проведенное на популяции из 16 888 человек с РПЖ, подтвердило статистически значимую связь между АДТ (включая ее продолжительность) и БА. Из исследования исключались мужчины, получающие химиотерапию, т.к. она может приводить к когнитивной дисфункции и, соответственно, быть причиной статистических ошибок. Из пациентов с деменцией в исследование были включены только те, у кого диагноз был установлен после начала АДТ. Большие когорты пациентов, страдающих РПЖ, содержат гетерогенную популяцию, включая людей с различными стадиями рака, а также сопутствующие факторы, такие как болевой синдром, химиотерапию, психосоциальный и эмоциональный стресс. Все эти факторы должны подлежать обязательному учету, т.к. способны самостоятельно приводить к развитию БА [71][72] и быть причиной ошибочных выводов.

В рассмотренных нами исследованиях не учитывались уровни эстрадиола и ИФР-1, которые способны значительно влиять на снижение памяти. Использование различных методологий АДТ также оказывает большое влияние на возникновение ошибочных результатов. Так, например, у мужчин с РПЖ, получавших АДТ лучевым методом, не было обнаружено снижения когнитивных функций [56]. АДТ может проводиться с использованием различных методик, включая двустороннюю орхиэктомию или медикаментозное лечение с применением агонистов гонадотропин-рилизинг-гормона (ГнРГ), антиандрогенов, а также возможна комбинированная терапия [47]. Соответственно, различные формы АДТ оказывают разное влияние на гипоталамо-гипофизарно-гонадную ось, что находит свое отражение в результатах исследований.

J.H. Hong и соавт. обнаружили, что снижение когнитивных функций было более выражено у пациентов, получавших антиандрогенную терапию, чем у тех, кто перенес комбинированную андрогенную блокаду, двустороннюю орхиэктомию и принимал ГнРГ [47]. У мужчин, получавших андрогенную блокаду по поводу РПЖ, были выявлены значительное повышение плазменных уровней бета-амилоида, а также повышенная тревожность и симптомы депрессии [17].

Факторы, влияющие на когнитивные функции, ассоциированные с проведением АДТ, могут также включать настроение и усталость, особенно у пациентов, чье заболевание с большой вероятностью приводит к смерти [73]. У пожилых людей достаточно сложно проводить оценку снижения когнитивных функций, т.к. оно может быть обусловлено возрастными изменениями.

## ВЛИЯНИЕ ТЕРАПИИ ТЕСТОСТЕРОНОМ НА КОГНИТИВНЫЕ ФУНКЦИИ У ПАЦИЕНТОВ С БА

Влияние терапии Т на улучшение когнитивных функций и снижение прогрессирования БА исследовалось в 17 исследованиях, которые мы обобщили в таблице 2 [74–90]. Некоторые исследования показали положительно влияние Т-терапии наопределенные домены когнитивных функций у здоровых и гипогонадных пожилых мужчин [75][78–80][85][88–90], в то время как остальные не давали однозначных результатов [74][76][81][82][84][87].

Большинство исследований, в которых не было выявлено улучшения когнитивных функций, были проведены на относительно здоровом населении (60–65 лет) с сексуальными, но не когнитивными нарушениями. S.M. Resnik и соавт. исследовали популяцию из 788 мужчин 65 лет с сексуальными нарушениями [74]. Авторы не обнаружили корреляции между проведением Т-терапии и когнитивными функциями. Лечение состояло из применения Т-геля в течение 90 дней с целью восстановления физиологического уровня Т в плазме крови. В исследовании G. Huang и соавт. использование Т-геля у мужчин 60 лет с низким содержанием Т в плазме не приводило к улучшению памяти [76]. P.R. Asih и соавт. получили аналогичные результаты у 61-летних мужчин при трансдермальном введении Т [77]. M.H. Emmelot-Vonk и соавт. исследовали здоровых мужчин через 6 и 36 недель перорального приема 80 мг Т ундеканоата и не отметили какого-либо улучшения когнитивных функций [81]. M.M. Cherrier и соавт. при оценке небольшой группы мужчин с гипогонадизмом обнаружили лишь умеренное улучшение вербальной памяти.

Т-терапия включает в себя широкий диапазон доз, варьирующихся от пути введения: трансдермальный (гель 7,5 г 1%), пероральный (80 мг/сут) и внутримышечный (200 мг/нед). Все это находит отражение в результатах исследований.

P.M. Maki и соавт. обнаружили, что Т-энантат (при приеме 200 мг каждые 2 нед) снижает вербальную память у мужчин [83]. Однако число пациентов в данном исследовании было ограничено (15 испытуемых), что говорит о низкой достоверности его результатов. Различные систематические обзоры показали, что низкий уровень Т в плазме крови может коррелировать со снижением когнитивных функций у здоровых и гипогонадных мужчин [91–93].

G. Verdile и соавт. исследовали 427 мужчин с когнитивными нарушениями и обнаружили, что концентрации ЛГ и свободного Т имеют обратную корреляцию с уровнем бета-амилоида плазмы крови, а также с отложением амилоида в головном мозге [94]. Посмертный гистопатологический анализ ткани головного мозга у женщин в постменопаузе не выявил изменений в уровнях андрогенов и эстрогенов. Напротив, у женщин с БА уровни эстрогенов и андрогенов были низкими, вне зависимости от возраста пациенток. В головном мозге мужчин возраст имеет корреляцию со снижением уровня андрогенов и эстрогенов. У людей с прогрессирующей БА уровни Т, но не эстрогена, в головном мозге были значительно снижены [95]. Примечательно, что у пациентов с потерей памяти уровень бета-амилоида коррелировал с общим и свободным уровнями Т [96].

## ОБСУЖДЕНИЕ

На животных моделях было продемонстрировано явное влияние Т на снижение отложения бета-амилоида в головном мозге и риска развития БА соответственно. Однако результаты клинических исследований являются противоречивыми.

Большинство исследований показали, что АДТ у пациентов с РПЖ снижает когнитивные функции и увеличивает риск развития болезни Паркинсона. Однако не все методы АДТ имеют одинаковый эффект. Антиандрогенные препараты имеют наиболее выраженный отрицательный эффект, в то время как агонисты ГнРГ, по-видимому, имеют меньшую вовлеченность в процесс ухудшения когнитивных функций. Онкоурологи должны учитывать это клинический аспект при выборе начальной тактики лечения.

Многие клинические исследования показали, что субъекты с низким уровнем андрогенов подвергаются более высокому риску снижения когнитивных функций [97], потери памяти, дефицита внимания и двигательной функции при рассеянном склерозе [97–99] и БА [85]. Прогрессирующее снижение сывороточного уровня ЛГ и Т на ранней доклинической стадии может считаться прогностическом признаком БА [94]. Большинство исследований отражают, что физиологическая концентрация Т в плазме необходима для поддержания нормальной функции головного мозга, а его снижение предрасполагает к развитию деменции и БА, свободный Т, в свою очередь, может приводить к снижению когнитивных функций и повышенному риску БА [100]. Наиболее значимые когортные исследования показали, что проведение АДТ у мужчин с РПЖ коррелировало с более высокой заболеваемостью БА [47–50][54]. Методика выполнения АДТ имеет важное значение, т.к. оказывает различное клиническое воздействие. Можно предположить, что андрогены играют защитную роль в поддержании структурной и функциональной целостности нейронов. Нейропротекторный эффект Т осуществляется на клеточном уровне за счет повышения функциональной активности митохондрий и улучшения клеточной биоэнергетики [22]. Экспрессия АР, ЭР и ароматазы заметно снижается у мужчин с гипогонадизмом и сахарным диабетом 2 типа, однако заместительная Т терапия полностью устраняет эти дефициты [101].

Тем не менее, эффекты от введения Т пациентам с БА противоречивы. Учитывая значительные расхождения в результатах различных исследований важно обеспечить более глубокое понимание методологических различий между ними. Один из наиболее критических аспектов в методологии представлен дозой введения Т и приверженностью к терапии. Приверженность необходима для поддержания стабильного уровня гормона в плазме.

Плазменный уровень свободного Т и 17β-эстрадиола необходим для оценки эффекта терапии. После введения Т важно определить его метаболиты (17β-эстрадиол, ДГТ), т.к. они участвуют в поддержании функционирования нейронов. Даже если пациенты получают одинаковую Т-терапию, у них может наблюдаться различный клинический эффект из-за различной абсорбции и метаболизма Т (рис. 1).

В большинстве исследований эффекты от введения Т оценивались у здоровых и гипогонадных мужчин, и только в нескольких оценивалось влияние на пациентов с БА [85][102]. По результатам обоих исследований было выявлено клиническое улучшение.

Помимо Т, в качестве нейрорегенерационной терапии могут использоваться другие андрогены (оксандролон, станозолол, нандролон и т.д.), а также селективные модуляторы андрогенных рецепторов, которые оказывают нейропротекторное действие при БА [103], но требуют проведения дополнительных исследований.

Отсутствие корреляции между Т и БА в некоторых исследованиях может объясняться обратными этиологическими механизмами, статистическими ошибками, а также ненадлежащим учетом факторов индивидуальности.

## ЗАКЛЮЧЕНИЕ

Хотя между исследованиями существует некоторое клиническое несоответствие, андрогены оказывают значительное влияние на функцию головного мозга и полезны для пациентов с БА. Низкие уровни циркулирующих андрогенов следует рассматривать как существенный фактор риска развития БА и потери памяти. При сниженном уровне Т в плазме мужчин его введение способствует повышению когнитивной работоспособности и памяти, лечение следует начинать на ранней стадии заболевания. У мужчин и женщин с БА андрогены улучшают психическое состояние и замедляют прогрессирование заболевания, оказывая протективное действие.

В будущем необходимо проведение исследований на большой популяции с учетом факторов индивидуальности и более конкретным подходом к оценке когнитивных функций и причинно-следственной связи введения Т при БА.

## ДОПОЛНИТЕЛЬНАЯ ИНФОРМАЦИЯ

Источники финансирования. Работа выполнена по инициативе авторов без привлечения финансирования.

Конфликт интересов. Авторы декларируют отсутствие явных и потенциальных конфликтов интересов, связанных с содержанием настоящей статьи.

Участие авторов. Кузнецов К.О. — разработка концепции и дизайна исследования, получение и анализ данных, интерпретация результатов; Хабибуллина Р.Х. — разработка дизайна исследования, написание статьи; Хайдарова Р.Р. — разработка дизайна исследования, написание статьи; Стыценко Е.С. — анализ данных, написание статьи; Философова В.И. — интерпретация результатов, написание статьи; Нуриахметова И.Р. — получение и анализ данных, редактирование статьи; Хисамеева Э.М. — интерпретация результатов, редактирование статьи; Важоров Г.С. — анализ данных, редактирование статьи; Хайбуллин Ф.Р. — получение данных, редактирование статьи; Иванова Е.А. — интерпретация результатов; Горбатова К.В. — написание и редактирование статьи. Все авторы внесли равный вклад в написание статьи и одобрили ее финальную версию перед публикацией, выразили согласие нести ответственность за все аспекты работы, подразумевающую надлежащее изучение и решение вопросов, связанных с точностью или добросовестностью любой части работы.
